# *In vitro* antibacterial activity of tigecycline against multidrug-resistant bacteria isolated at the Sourô Sanou University teaching hospital in Bobo-Dioulasso, Burkina Faso

**DOI:** 10.4102/ajlm.v14i1.2895

**Published:** 2025-12-18

**Authors:** Odilon D. Kaboré, Mélissa J. Gonfouli, Merci Muhigwa, Abdourahmane Sow, Fernand Michodigni, André Nagalo, Jacques Zoungrana, Arsène Hema, Augustin Konkobo, Hervé Kafando, Adama Ouattara, Armel Poda, Sylvain Godreuil, Abdoul-Salam Ouedraogo

**Affiliations:** 1 Laboratory of Bacteriology and Virology, Sourô Sanou University Teaching Hospital, Bobo-Dioulasso, Burkina Faso; 2 Burkina Faso National Reference Laboratory for Antimicrobial Resistance, Bobo-Dioulasso, Burkina Faso; 3 Laboratory of Emerging and Re-emerging Pathogens, School of Health Sciences, Nazi Boni University, Bobo-Dioulasso, Burkina Faso; 4 Higher Institute of Health Sciences, Nazi Boni University, Bobo-Dioulasso, Burkina Faso; 5 Region Wide Laboratory Services, West African Health Organization, Bobo-Dioulasso, Burkina Faso; 6 Department of Infectious and Tropical Diseases, Sourô Sanou University Teaching Hospital, Bobo-Dioulasso, Burkina Faso; 7 Department of Quality, Sourô Sanou University Teaching Hospital, Bobo-Dioulasso, Burkina Faso; 8 Centre Muraz, Bobo-Dioulasso, Burkina Faso; 9 Division of Urology, Sourô Sanou University Teaching Hospital, Bobo-Dioulasso, Burkina Faso; 10 Joint Research Unit, Research Institute for Development, National Center for Scientific Research, Inserm, Faculty of Infectious Diseases and Vectors: Ecology, Genetics, Evolution and Control, University of Montpellier, Montpellier, France

**Keywords:** *Acinetobacter baumannii*, Burkina Faso, Enterobacterales, extended-spectrum *β*-lactamase-producing Enterobacterales, in vitro, methicillin-resistant *Staphylococcus aureus*, multidrug-resistant bacteria, tigecycline

## Abstract

**Background:**

Tigecycline, an antibiotic effective against multidrug-resistant bacteria (MDRB), remains inaccessible in Burkina Faso’s hospitals for urgent care. Given the resulting therapeutic challenges and mortality in emergency services, evidence-based study of tigecycline’s efficacy on local bacterial clinical isolates is necessary before recommending its use.

**Objective:**

This study aimed to evaluate the activity of tigecycline on MDRB isolates at the Sourô Sanou University Teaching Hospital laboratory, Burkina Faso.

**Methods:**

This was a cross-sectional study with prospective and consecutive sampling of MDRBs. The latter included extended-spectrum *β*-lactamase-producing Enterobacterales (ESBL-E), carbapenem-resistant strains (CRS), and methicillin-resistant *Staphylococcus aureus* (MRSA), which were isolated from urine, blood, pus and puncture fluids between 01 June 2022 and 31 August 2022. Antimicrobial susceptibility testing was conducted using the modified Kirby-Bauer method, and the results were interpreted according to the standard set by the European Committee on Antimicrobial Susceptibility Testing in 2021.

**Results:**

A total of 117 MDRBs, including 93 Enterobacterales, 15 carbapenem-resistant *Acinetobacter baumannii* (CRAB), and 9 MRSAs were collected. The proportion of ESBL-E was 58% (68/117), followed by CRS (34%, 40/117) and MRSA (8%, 9/117). The activity of tigecycline was 95.5% (43/45) on ESBL-E, 72.5% (29/40) on CRS (including 10/15 *CRAB*), and 89% (8/9) on MRSA.

**Conclusion:**

The activity of tigecycline was highly effective on ESBL-E, carbapenem resistant Enterobacterales and MRSA, and moderate on *CRAB*.

**What this study adds:**

This was the first report on the evaluation of tigecycline activity on MDRBs in Burkina Faso. This non-marketed antibiotic in Burkina Faso could represent an alternative to spare carbapenems in the treatment of ESBL-E infections, and a last resort antibiotic against susceptible CRS infections in Burkina Faso’s hospitals.

## Introduction

Antimicrobial resistance (AMR) has emerged as one of the leading public health threats of the 21st century, and poses an economic burden to the healthcare system because of long hospitalisations and higher morbidity and mortality.^1^ In 2019, there were approximately 4.95 million deaths associated with antimicrobial resistance, including 1.27 million attributable to multidrug-resistant bacteria (MDRB). The low-income countries of sub-Saharan Africa are the most affected, with an estimated mortality of 27.3 deaths per 100 000 inhabitants.^2^

The most problematic MDRBs encompass extended-spectrum β-lactamases (ESBL)-producing Enterobacterales (ESBL-E), carbapenemase-producing Enterobacterales (known as carbapenem-resistant Enterobacterales, carbapenem-resistant *Acinetobacter baumannii* (CRAB), and *Pseudomonas aeruginosa*. In addition to vancomycin-resistant enterococcus and methicillin-resistant *Staphylococcus aureus* (MRSA), they constitute a class of ‘superbugs’ called ESKAPE, an acronym with dual significance reflecting both the first letter of the bacterial name and their ability to evade the bactericidal effects of antibiotics.^3,4^

Given the global surge in ESBL-E and carbapenem-resistant organisms, it is imperative to find alternative treatments to carbapenems and develop novel therapeutic agents to combat infections caused by these resistant bacteria.

New antibiotics have been introduced into the market of developed countries over the last two decades to improve the treatment regimens of MDRB infections,^5,6,7^ but remain inaccessible in low- and middle-income countries affected by high rates of therapeutic deadlocks linked to superbug infections.^2,8^ One of these new antibiotics is tigecycline, a semisynthetic 9-tertbutyl-glycylamido derivative of minocycline, which acts by higher binding affinity to the 30S ribosomal subunit of bacteria and by blocking entry of amino-acyl tRNA molecules into the A site of ribosomes. Amino acid residues are prevented from becoming incorporated into elongating peptide chains, thus leading to inhibition of protein synthesis. In addition, the glycylamido side chain at the 9th position on the D-ring results in enhanced activity and allows the molecule to resist efflux pumps compared to classical tetracyclines.^9^ This antibiotic inhibits the growth of multiple resistant Gram-positive, Gram-negative and anaerobic bacteria, and atypical bacteria, including MRSA and ESBL-E, but it remains vulnerable to the chromosomally-encoded multidrug efflux pumps of *Pseudomonas aeruginosa* and Proteae sub-species.^10,11^ In 2005, tigecycline was approved by the US Food and Drug Administration (FDA),^12,13^ and the European Commission granted a valid marketing authorisation throughout the European Union for tigecycline (TYGACIL^®^) on 24 April 2006.^14^ Despite being available in developed countries and proven effective against MDRB in many studies, this life-saving antibiotic remains inaccessible for urgent treatments in the hospitals of Burkina Faso, resulting in preventable deaths caused by therapeutic deadlocks.^5,12,15^

In this regard, the current priority for the hospitals in Burkina Faso is to find alternatives to reduce the overuse of carbapenems in ESBL-E infections, while exploring effective antibiotic regimens to treat carbapenem-resistant organisms in therapeutic deadlock contexts. This study aimed to evaluate the activity of tigecycline on MDRB isolated from clinical samples at the referral healthcare centre in Bobo-Dioulasso. The expected results will allow us to authorise the supply of tigecycline in the reserve antibiotics for the treatment of patients in the therapeutic deadlocks.

## Methods

### Ethical considerations

The study was conducted as part of the routine diagnosis of bacterial infections under the authorisation of the Ethics Committee for Research at the National Reference Laboratory for Antimicrobial Resistance (approval number: N°2022-06-053MSHP/SG/CHUSS/DG/DL/SBV/LNR-RAM), in line with the Declaration of Helsinki. The anonymity of the participants involved in the study was maintained in this study. Only the laboratory number was mentioned to guarantee confidentiality. No specific declaration of informed consent was required from the patients, as the experiments were part of the routine diagnosis supervised by the Burkina Faso Ministry of Health and Public Hygiene, which has the authority to process patient data for public health surveillance purposes. The outcomes of this work will be used to improve antibiotic prescriptions in patients with MDRB in Burkina Faso’s hospitals.

### Study design, period, population and site

This was a cross-sectional study with a prospective design that used consecutive sampling of MDRB clinical isolates ESBL-E, carbapenem-resistant strains (CRS) and MRSA from patients between 01 June 2022 and 31 August 2022. Bacteriological analysis was carried out at the bacteriology laboratory of Sourô Sanou University Teaching Hospital, Bobo-Dioulasso. This laboratory hosts the Burkina Faso National Reference Laboratory for Antimicrobial Resistance.

### Data and clinical samples collection

The participants’ demographic, clinical service and sample types data were collected. All patients whose samples were positive for ESBL, CRS, and MRSA phenotypes, except *P. aeruginosa* and members of the Proteae tribe (*Proteus* spp., *Providencia* spp. and *Morganella morganii*) which are intrinsically resistant to tigecycline,^13^ were enrolled. The samples consisted of urine, pus, puncture fluids, and blood.

### Isolation, identification and antimicrobial susceptibility testing

#### Media used for bacterial isolation

Regarding the isolation of MDRBs from urine samples, the culture media employed in this process were cystine lactose electrolyte deficient agar, and eosin methylene blue agar. Considering bacterial isolation from blood culture, pus, and serous fluid cultures, appropriate agar plates (enriched chocolate, eosin methylene blue or Chapman agar) were selected based on the presumed bacterial characteristics from a Gram stain.

#### Bacterial identification

Bacterial presumptive identification was based on their morphological and cultural characteristics. Enterobacterales and non-fermenting Gram-negative bacilli were identified using commercialised kits, analytical profile index (API) 20E and API 20 NE strips (bioMérieux, Marcy-l’Étoile, France), respectively.^16^ For Gram-positive cocci, catalase and coagulase tests were used. Briefly, the catalase test was conducted using the slide method with 1 drop of 3% hydrogen peroxide to observe an immediate bubble formation, indicating the presence of the catalase enzyme. Regarding the coagulase test, a lump of bacterial cells was mixed into a drop of ethylenediaminetetraacetic acid-treated rabbit plasma on a microscope slide to observe the clumping of bacterial cells within 10 seconds. When a sample of Gram-positive cocci responded positively to the catalase and coagulase tests, the microorganism was confirmed as *S. aureus*.

#### Antimicrobial susceptibility testing

Susceptibility of each isolate to various antimicrobial agents was determined through the Kirby-Bauer (KB) method (disc-diffusion method)^17^ on Mueller-Hinton Agar (MHA) plates (Biomaxima, Lublin, Poland). Briefly, single colonies of each clinical isolate were suspended in 5 mL sterile distilled water. The turbidity of the suspension was adjusted to 0.5 McFarland’s standard and subsequently spread onto the surface of the MHA plate using a sterile cotton swab. Afterwards, antibiotic-impregnated disks were carefully placed onto an MHA plate using forceps, and the plates were then incubated at 37 °C overnight. The inhibition diameters of 15 µg tigecycline were measured and interpreted as resistant or susceptible using standard reference values according to CA-SFM-EUCAST 2021.^18^

### Phenotypic antibiotic-resistance analysis

#### Extended-spectrum β-lactamase detection using the double synergy test

The double synergy test was used for the detection of ESBL production. Briefly, it consisted of placing a 30 µg disk of third-generation cephalosporin (cefotaxime, ceftriaxone, ceftazidime) and/or other (aztreonam [30 µg] or cefepime [30 µg] at a distance of 30 mm, centre to centre, of an amoxicillin/clavulanic acid disk (20 µg/10 µg). The production of ESBL resulted in very characteristic synergistic images between the third-generation cephalosporin disk or aztreonam (30 µg) and amoxicillin/clavulanic acid disk (20 µg/10 µg): a so-called champagne cork image.^18^

#### Carbapenem-resistant strains detection

Regarding the detection of carbapenem-resistant Enterobacterales, any isolate with decreased susceptibility to ertapenem (inhibition diameter < 25 mm; 10 µg) through agar diffusion test was considered resistant. For *A. baumannii*, a decreased susceptibility to imipenem 10 µg (inhibition diameter < 21 mm) or meropenem 10 µg (inhibition diameter < 15 mm) through agar diffusion test was considered resistant.^18^

#### Methicillin-resistant *S. aureus* detection

For MRSA identification, strains with a cefoxitin (30 µg disk) zone diameter < 22 mm were considered as MRSA phenotype.^18^

### Activity of tigecycline on multidrug-resistant bacteria detection

We tested the activity of tigecycline (15 µg) on all MDR strains of *Escherichia coli* in accordance with the EUCAST 2021 guidelines, and an inhibition diameter ≥ 18 mm was considered sensitive to tigecycline. For the other species of Enterobacterales, the inhibition diameter ≥ 19 mm has been considered for susceptible strains as suggested by the FDA (https://www.accessdata.fda.gov/drugsatfda_docs/label/2013/021821s026s031lbl.pdf). All MRSA strains having an inhibition diameter ≥ 19 mm were considered susceptible to tigecycline.^18^ Considering CRAB, susceptibility to tigecycline was inferred using the clear zone diameters suggested by the FDA criteria.^19^ Susceptibility to tigecycline was defined based on clear zone diameters as susceptible (≥ 19 mm), whereas clear zone diameters < 19 mm were considered as resistant, although FDA guidelines suggest intermediate for 15 mm – 18 mm, or ≤ 14 mm for resistant.^19^

### Quality controls

Quality control of antimicrobial susceptibility testing was assessed by using reference strains such as *E. coli* ATCC 25922, *S. aureus* ATCC 25929, and *Klebsiella pneumoniae* ATCC 700603, in accordance with EUCAST guidelines.^18^

### Data processing and analysis

Data were recorded in a Microsoft Excel 2016 worksheet, which was used to create graphs and tables. Statistical analysis was performed with Epi Info 7™ software. The qualitative variables were expressed in proportions, whereas the quantitative variables were expressed in means with their 95% confidence intervals. Proportions of MDRBs sensitive or resistant to tigecycline were compared with the Pearson Chi-square test when there was a normal distribution; otherwise, the Fisher’s exact test was used. The significance threshold of 5% was considered for all statistical tests.

## Results

**FIGURE 1:**
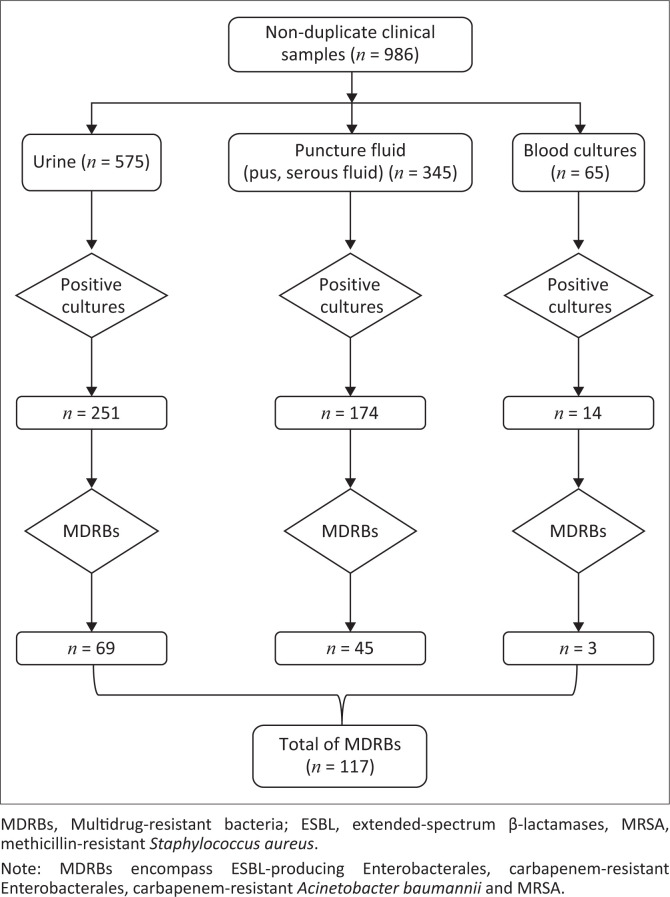
Distribution of non-duplicate clinical samples and multidrug-resistant bacteria (*n* = 117) isolated, Sourô Sanou University Teaching Hospital in Bobo-Dioulasso, Burkina Faso, 01 June 2022 – 31 August 2022.

A total of 986 non-duplicate samples were included during the study period (01 June 2022 to 31 August 2022). The bacteriological yield was 44.52% (439/986) positive cultures. Amongst the 439 positive cultures, 117 strains were MDRBs (26.65%), isolated from 117 patients (Figure 1).

### Patient sociodemographic characteristics

Out of the 117 patients observed with MDRBs, the male gender was predominant (53.8%). The average age of the 117 patients from whom we isolated MDRBs was 41.27 ± 17.9 years, ranging from 1 month to 85 years. The most represented age group was 40–50 years (17.9%), followed by 20–30 years (14.5%).

Regarding the distribution of MDRBs by patient origin, 80% came from various hospitalisation wards of the Sourô Sanou University Teaching Hospital, and 20% from other health facilities in the city of Bobo-Dioulasso. Considering the hospitalisation wards, the urology department was most represented with 14.5%, followed by the intensive care unit (12%).

### Distribution of multidrug-resistant bacteria (*n* = 117) according to bacterial species

**FIGURE 2:**
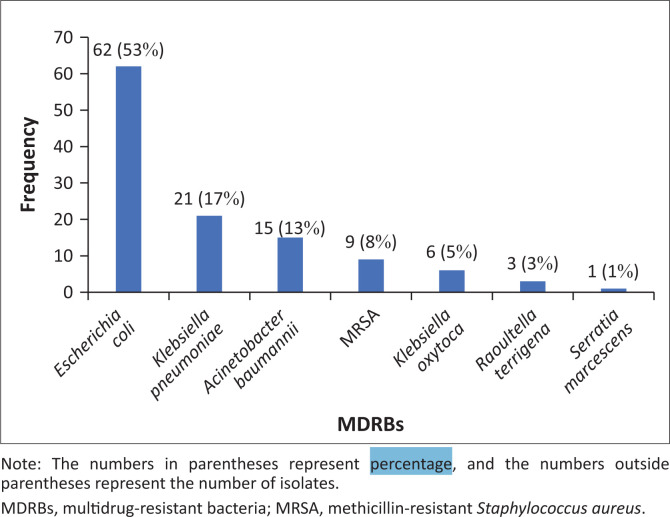
Distribution of multidrug-resistant bacteria according to bacterial species, Sourô Sanou University Teaching Hospital in Bobo-Dioulasso, Burkina Faso, 01 June 2022 – 31 August 2022 (*N* = 117).

Out of the 117 MDR isolates, *E. coli* was the most represented bacterial species (*p* < 0.018) with a frequency of 53% (62/117), followed by *K. pneumoniae* accounting for 17% (20/117). As described in Figure 2, CRAB was isolated with a frequency of 13%, MRSA at 9%, and *K. oxytoca* at 5%.

### Distribution of multidrug-resistant bacteria according to resistance phenotypes

Out of the 117 MDRBs isolated, the most predominant phenotype was ESBL-producing Enterobacterales (58%), including *E. coli* (72.58%), followed by *K. pneumoniae* with 67%. The resistance phenotypes to carbapenems (CRS) represented 37% (*p* < 0.001; ESBL-E versus CRS). All strains of *A. baumannii* (*n* = 15) were resistant to carbapenems. For MRSA, nine strains were isolated, as detailed in Table 1.

**TABLE 1:** Identification and distribution of multidrug-resistant bacteria according to resistance phenotypes, Sourô Sanou University Teaching Hospital in Bobo-Dioulasso, Burkina Faso, 01 June 2022 – 31 August 2022 (*N* = 117).

Bacteria	ESBL		CRS		MRSA		Total
	*n*	%	*n*	%	*n*	%	
**Enterobacterales**	-	-	-	-	-	-	93
*Escherichia coli*	45	72.58	17	27.42	-	-	62
*Klebsiella pneumonia*	14	67.00	7	33.00	-	-	21
*Klebsiella oxytoca*	6	100.00	0	0.00	-	-	6
*Raoultella terrigena*	3	100.00	0	0.00	-	-	3
*Serratia* spp.	0	0.00	1	100.00	-	-	1
**Non-fermenting Gram negative**							
*Acinetobacter baumannii*	0	0.00	15	100.00	-	-	15
**Gram positive**							
*Staphylococcus aureus*	-	-	-	-	9	100	9
**Total**	**68**	**58.00**	**40**	**34.00**	**9**	**8**	**117**

Note: Significance of bold values represent the distribution of bacterial phenotype (ESBL, CRS and MRSA). The most predominant phenotype (*p* < 0.001) was ESBL-producing Enterobacterales (58%) compared to CRS.

ESBL, extended-spectrum β-lactamase-producing; CRS, carbapenem-resistant strains; MRSA, methicillin-resistant *Staphylococcus aureus*.

### *In vitro* activity of tigecycline on multidrug-resistant bacteria species

Nine strains of Enterobacterales, including 6 (66.67%) *E. coli* (2 ESBLs and 4 CRS) and 3 (33.33%) *K. pneumoniae* (1 ESBL and 2 CRS), exhibited resistance to tigecycline. Other enterobacteria, *K. oxytoca, Raoultella terrigena*, and *Serratia marcescens*, were all susceptible to tigecycline. Considering the strains of *A. baumannii*, 66.67% (10/15) were susceptible to tigecycline. There was no statistically significant difference in the distribution of *in vitro* activity of tigecycline on MDRBs species (*p* > 0.32), as detailed in Table 2.

**TABLE 2:** Tigecycline activity according to multidrug-resistant bacteria species, Sourô Sanou University Teaching Hospital in Bobo-Dioulasso, Burkina Faso, 01 June 2022 – 31 August 2022.

Bacterial strains	Resistant†		Susceptible†		Total
	*n*	%	*n*	%	
**Enterobacterales**	-	-	-	-	-
*Escherichia coli*	6	9.67	56	90.32	62
*Klebsiella pneumoniae*	3	14.28	18	85.71	21
*Klebsiella oxytoca*	0	0.00	6	100.00	6
*Raoultella terrigena*	0	0.00	3	100.00	3
*Serratia marcescens*	0	0.00	1	100.00	1
**Non-fermenting Gram negative**					
*Acinetobacter baumannii*	5	33.30	10	66.00	15
**Gram positive**					
*Staphylococcus aureus*	1	11.10	8	88.80	9

ESBL, extended-spectrum β-lactamase-producing; CRS, carbapenem-resistant strains; MRSA, methicillin-resistant *Staphylococcus aureus*.

† , Resistant and susceptible bacterial strains: *p* > 0.32.

### Tigecycline activity against resistance phenotypes

Out of 117 antibiotic-resistant bacterial isolates, the overall activity of tigecycline on all MDRBs was 95.33% (102/117) with *p*-value < 0.002. Out of the 68 ESBL-E strains, tigecycline was active against 65 (95.59%) of total strains. Considering the 40 CRS strains, 29 (72.50%) were susceptible to tigecycline. Of the nine MRSA isolated, only one strain was resistant. The activity of tigecycline against resistance phenotypes were described in Table 3.

**TABLE 3:** Activity of tigecycline against resistance phenotypes, Sourô Sanou University Teaching Hospital in Bobo-Dioulasso, Burkina Faso, 01 June 2022 – 31 August 2022 (*N* = 117).

Phenotypes	Resistant (*n* = 15)		Susceptible (*n* = 102)		Total	*p*-value
	*n*	%	*n*	%		
ESBL	3	4.41	65	95.59	68	< 0.002
CRS	11	27.50	29	72.50	40	
MRSA	1	11.11	8	88.88	9	

ESBL, extended-spectrum β-lactamase-producing; CRS, carbapenem-resistant strains; MRSA, methicillin-resistant *Staphylococcus aureus*.

### Tigecycline activity on the two predominant isolates of Enterobacterales (*E. coli* and *Klebsiella* spp.) resistance phenotypes

**FIGURE 3:**
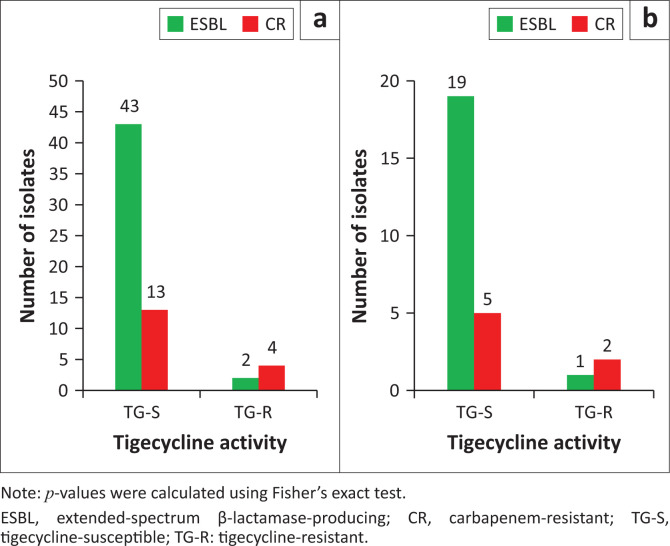
Tigecycline activity against (a) *Escherichia coli* resistance phenotypes (*n* = 62; *p* < 0.04) and (b) *Klebsiella* spp. resistance phenotypes (*n* = 27; *p* < 0.1), Sourô Sanou University Teaching Hospital in Bobo-Dioulasso, Burkina Faso, 01 June 2022 – 31 August 2022.

Regarding the ESBL-producing *E. coli* clinical isolates (45 strains) and the 17 carbapenem-resistant *E. coli* strains, tigecycline was active in 96% ESBL strains (43/45), while 76% CRS were found to be susceptible to tigecycline (13/17), *p* < 0.04 (Figure 3).

Considering the *Klebsiella* spp. clinical isolates (27 strains), and the 7 carbapenem-resistant of *K*. strains, tigecycline was active on 20 ESBL *Klebsiella* spp. (14 *K. pneumoniae* and 6 *K. oxytoca*) while 71% carbapenem-resistant *K. pneumoniae* have been found to be susceptible to tigecycline (5/7), *p* < 0.1 (Figure 3).

## Discussion

Limited treatment options for MDRBs represent a serious global health issue, particularly in low- and middle-income countries that struggle to access new antibiotics to overcome emerging CRS, ESBL-E, and MRSA infections.^8^ In this context, we carried out this study to investigate the *in vitro* activity of tigecycline against MDRBs and to provide local data to advocate for the availability of tigecycline in the reserve antibiotics for the management of therapeutic impasses.

Regarding the ESBL-E, a high rate of tigecycline activity (96%, 65/68) was observed in this study. This is similar to that reported in a systematic review in which tigecycline was microbiologically active against the majority of ESBL-E, including more than 99% of 1936 resistant *E. coli* isolates.^20^ In addition, our results were consistent with those of Zhang et al.^21^ in a global study on antimicrobial susceptibility between 2012 and 2016. The authors observed good activity of tigecycline on 98.4% of *E. coli* and 90.4% of *Serratia* spp.^21^ Furthermore, our observation was comparable with the finding reported by Morosini et al. in Spain (93.7%),^22^ but higher than the one mentioned by Bazi et al. for the ESBL-E (76%).^23^ Globally, tigecycline has excellent *in vitro* activity against almost all ESBL-E.^20^ This property allows it to evade many common resistance mechanisms, making it a suitable alternative for the treatment of many serious and life-threatening infections for which other antibiotics are no longer appropriate.^24^ As a result, tigecycline might circumvent the overuse of carbapenems during the era of MDR in ESBL-E in Burkina Faso’s hospitals, as demonstrated in a previous study in Thailand.^25^

Considering carbapenem-resistant Gram-negative bacteria, we observed a susceptibility of 72.5% (29/40) to tigecycline. This finding is higher than that reported by Bazi et al. in Morocco,^23^ and others in Réunion Island.^26^ The authors in Morocco found an overall susceptibility rate of 57% of carbapenem-resistant Gram-negative bacteria to tigecycline, and those in Réunion, 67%. These differences could be explained by the long-term routine use of tigecycline in these countries compared to Burkina Faso, where it has not yet been marketed. Indeed, carbapenem-resistant Gram-negative bacteria employ several resistance mechanisms, including decreased expression of outer membrane porins, modification of the tigecycline’s target site, and overexpression of efflux pumps to inactivate tigecycline.^27^ Therefore, the strength of the selection pressure for tigecycline would be an important parameter contributing to the complexity of resistance evolution in localities where it is widely used.^28^

Regarding carbapenem-resistant Enterobacterales, tigecycline activity was higher with carbapenem-resistant *E. coli* (13/17, 76%) than that observed with carbapenem-resistant *K. pneumoniae* (CRKP; 5/7, 71%), and this observation is supported by numerous studies.^29,30,31^ For example, Fatima et al. in Pakistan reported a better activity of tigecycline on 97% (34/35) for carbapenem-resistant *E. coli* versus 86.3% (88/102) for CRKP.^30^ The same trends were observed by Wang et al. in China, with significantly higher susceptibility rates of 97.73% for carbapenem-resistant *E. coli* compared to 64.86% for CRKP.^32^ Likewise, an increasing prevalence of CRKP isolates was reported from China to cause decreased tigecycline susceptibility.^33^ However, the lower susceptibility rates observed in our study compared to others may be because of our small sample size.^32^ Regarding the efflux-mediated mechanisms in CRKP, including high expression of the AcrAB-TolC and OqxAB efflux pumps, appears to play a key role in tigecycline resistance.^34,35^

In our study, the susceptibility rate for CRAB with tigecycline was 66%. This is similar to those reported by Bazi et al. (66%) in Morocco^23^ and by Navon-Venezia, Leavitt, and Carmeli (66%) in Israel.^36^ However, our observation was lower than that of Nahid et al. in South Africa. The authors reported a susceptibility of 75.8% to tigecycline on CRAB.^37^ The lower rate of the susceptibility that we found must be counterbalanced by the influence of culture media and testing technique, which often shows false-intermediate or false-resistant results in tigecycline susceptibility testing.^38^ Previous studies found some discrepancies between different antimicrobial susceptibility testing methods^39^ and the MHA composition (MHA from Oxoid versus MHA from Hopebio).^40^ Accordingly, broth microdilution is the reference method for testing tigecycline susceptibility, and a recent study reported that broth microdilution confirmed 91% susceptible CRAB strains to tigecycline yielded categorical agreement of 87.3% for CRAB with KB, suggesting that the KB method often shows false-resistant results.^38^ In addition, it has been reported that disk diffusion (used in our study) and E-test have a poor correlation with broth microdilution as the reference method, especially for *A. baumannii*. The content of manganese, zinc, and magnesium in different MHA may also explain these discrepancies in tigecycline inhibition diameters.^40,41^ However, caution should be taken in interpreting false resistance phenotypes through the disk diffusion assay in our study, which may exclude wrong inhibitory activity of tigecycline as the only therapeutic option in a field where multi-resistance is predominant.^42,43,44^ In cases of resistance, it would be advisable to carry out the antimicrobial susceptibility testing in a liquid medium using the VITEK-2 COMPACT (bioMérieux, Marcy-l’Étoile, France)^45^ to provide reliable results to the clinician.

Regarding the MRSA strains isolated in our study, we observed that one (1/9) strain was resistant to tigecycline (11%). This observation was consistent with the finding of Che Hamzah et al. in Terengganu, Malaysia, who found a sensitivity of 94% (85/90) to tigecycline on MRSA.^46^ By contrast, the susceptibility rate that our study found (89%) is lower than that observed by other authors who reported a high susceptibility of MRSA to tigecycline (> 99.9%) amongst 7098 *S. aureus* isolates.^47,48^ Similarly, Khalili et al. reported a susceptibility of 100% on 99 MRSA tested with the KB method in Tehran, Iran.^49^ However, the lower number of MRSA isolates (< 30) and the KB method used in our study may reduce the susceptibility rate, which does not allow us to draw a definitive conclusion about the activity of tigecycline on MRSA. Despite the relatively low rate of tigecycline susceptibility on MRSA in our study compared to others, resistance to tigecycline is barely found in *S. aureus* worldwide,^47,49,50^ and this molecule could be considered as an alternative antibiotic for most MRSA infections to decrease the risk of resistance to vancomycin, which remains the last-resort antibiotic for the treatment of MRSA infections.

### Strengths and limitations

This was the first study documented in Burkina Faso, that investigated tigecycline activity on MDRBs and could guide public health policy. This was a laboratory-based study with a limited sample size and locations. It should be supported by further clinical evaluations of the efficacy and adverse effects of tigecycline with multiple doses.

### Recommendations

The authors recommend that the Direction of Sourô Sanou University Teaching Hospital should make tigecycline available for treatment of infections caused by ESBL-E, carbapenem-resistant bacteria, or MRSA strains. We also request the hospital practitioners to use tigecycline as a reserve antibiotic to treat MDRB strains. We urge the other hospitals in the country to conduct their own studies to evaluate the effectiveness of tigecycline against clinical resistant bacteria (ESBL-E, CRS and MRSA) before considering it as a reserve antibiotic.

### Conclusion

In this current study, the activity of tigecycline was highly effective *in vitro* against ESBL-E and moderate against carbapenem-resistant Enterobacterales and CRAB and on MRSA. Therefore, tigecycline was recommended as an option for the treatment of MDRB infections in the hospitals of Burkina Faso. However, it should be used in combination therapies to mitigate resistance, a common drawback of monotherapy against CRS. Furthermore, the presence of resistance phenotypes amongst the MDRBs reported in this study, even before the official introduction of tigecycline in Burkina Faso’s hospitals, necessitates its usage as a reserve antibiotic. Finally, despite the FDA’s warning of an increased mortality risk associated with the use of tigecycline,^51^ this non-marketed antibiotic in Burkina Faso could represent a suitable therapeutic alternative to spare carbapenems in the treatment of ESBL-E infections.
